# Comparison of fMRI BOLD Response Patterns by Electrical Stimulation of the Ventroposterior Complex and Medial Thalamus of the Rat

**DOI:** 10.1371/journal.pone.0066821

**Published:** 2013-06-24

**Authors:** Pai-Feng Yang, You-Yin Chen, Der-Yow Chen, James W. Hu, Jyh-Horng Chen, Chen-Tung Yen

**Affiliations:** 1 Interdisciplinary MRI/MRI Lab, Department of Electrical Engineering, National Taiwan University, Taipei, Taiwan; 2 Department of Biomedical Engineering, National Yang-Ming University, Taipei, Taiwan; 3 Department of Psychology, National Cheng Kung University, Tainan City, Taiwan; 4 Faculty of Dentistry, University of Toronto, Toronto, Canada; 5 Neurobiology and Cognitive Science Center, National Taiwan University, Taipei, Taiwan; 6 Institute of Zoology, National Taiwan University, Taipei, Taiwan; National Yang-Ming University, Taiwan

## Abstract

The objective of this study was to compare the functional connectivity of the lateral and medial thalamocortical pain pathways by investigating the blood oxygen level-dependent (BOLD) activation patterns in the forebrain elicited by direct electrical stimulation of the ventroposterior (VP) and medial (MT) thalamus. An MRI-compatible stimulation electrode was implanted in the VP or MT of α-chloralose-anesthetized rats. Electrical stimulation was applied to the VP or MT at various intensities (50 µA to 300 µA) and frequencies (1 Hz to 12 Hz). BOLD responses were analyzed in the ipsilateral forelimb region of the primary somatosensory cortex (iS1FL) after VP stimulation and in the ipsilateral cingulate cortex (iCC) after MT stimulation. When stimulating the VP, the strongest activation occurred at 3 Hz. The stimulation intensity threshold was 50 µA and the response rapidly peaked at 100 µA. When stimulating the MT, The optimal frequency for stimulation was 9 Hz or 12 Hz, the stimulation intensity threshold was 100 µA and we observed a graded increase in the BOLD response following the application of higher intensity stimuli. We also evaluated c-Fos expression following the application of a 200-µA stimulus. Ventroposterior thalamic stimulation elicited c-Fos-positivity in few cells in the iS1FL and caudate putamen (iCPu). Medial thalamic stimulation, however, produced numerous c-Fos-positive cells in the iCC and iCPu. The differential BOLD responses and c-Fos expressions elicited by VP and MT stimulation indicate differences in stimulus-response properties of the medial and lateral thalamic pain pathways.

## Introduction

Nociceptive sensory processing within the thalamus involves 2 distinct and parallel systems: the lateral and medial systems [1]. Lateral regions of the thalamus project to the primary (S1) and secondary (S2) somatosensory cortices, which are considered important in the sensory-discriminative aspects of pain. Medial regions of the thalamus transmit processed nociceptive signals to the anterior cingulate cortex, insular cortex, and other limbic brain areas, which might be involved in the affective-motivational aspects of pain [2]–[5]. Most primary studies of these 2 systems used recording and tracing methods to investigate the anatomical and electrophysiological interconnections of these brain regions. However, it remains unclear if fundamental differences in signal processing exist between the medial and lateral thalamic pathways. A global comparison using direct stimulation and functional brain imaging is, therefore, needed.

Functional magnetic resonance imaging (fMRI) uses blood oxygenation level-dependent (BOLD) contrasts to correlate neural activities with neurovascular changes [6]–[8]. Although fMRI measures neural activity indirectly, it has the advantages of being noninvasive and capable of detecting whole-brain activation patterns. It is, therefore, a preferred method for evaluating human cognitive function. In addition, fMRI technique can be used for the investigation of functional changes of specific brain pathways in animal models. For examples, several studies combined fMRI with direct brain microstimulation to identify functional neural connections and BOLD responses, including in the visual system of the monkey [9], and hippocampal formation [10], the thalamocingulate pathway [11], and in Parkinson disease [12], [13].

To test the hypothesis that medial and lateral thalamic pathways have differential sensory processing properties, we monitored the BOLD responses in the forebrain following application of electrical stimulation to the ventroposterior (VP) or the medial (MT) thalamus. In addition, we investigated the functional activation areas associated with VP or MT stimulation by evaluating early-gene Fos expression in cortical and subcortical structures. Results indicated that the 2 thalamocortical pathways have distinct activation areas and c-Fos activation patterns, as well as different intensity- and frequency-dependent stimulation-response properties.

## Materials and Methods

All experimental procedures were approved by the Institutional Animal Care and Use Committee of National Taiwan University and adhered to guidelines established by codes for the experimental use of animals by the Council of Agriculture, Taiwan. In this study, all efforts were made in order to minimize both the number and suffering of the animals. Thirty female Long Evans rats weighing 250 g to 350 g were used, including 12 rats for fMRI experiments (6 for VP stimulation and 6 for MT stimulation), and 18 rats for c-Fos immunohistochemistry. Rats were kept in a 12-h dark/light cycle environment at a temperature of 22°C with food and water available ad libitum.

### Electrode Implantation and Recordings

A head mask was applied to each rat, providing an anesthetic of 4% isoflurane in 100% O_2_. A cannula was inserted into the rat femoral vein for administering anesthetic and the anesthetic was then changed to α-chloralose (80 mg/kg) and medetomidine hydrochloride (0.05 mg/kg). The addition of a low dosage of medetomidine (regular dose is 0.25–0.5 mg/kg) was to keep the rat under a suitable anesthesia level for surgery. On the basis of pharmacokinetic study of medetomidine hydrochloride in blood is about 1 hour [14]. We started deep brain stimulation 2 hr later. Therefore, there should be minimal lingering medetomidine effect during the stimulation experiment. After 1 h, a continuous infusion of α-chloralose was provided at a rate of 30 mg/kg/h.

**Figure 1:**
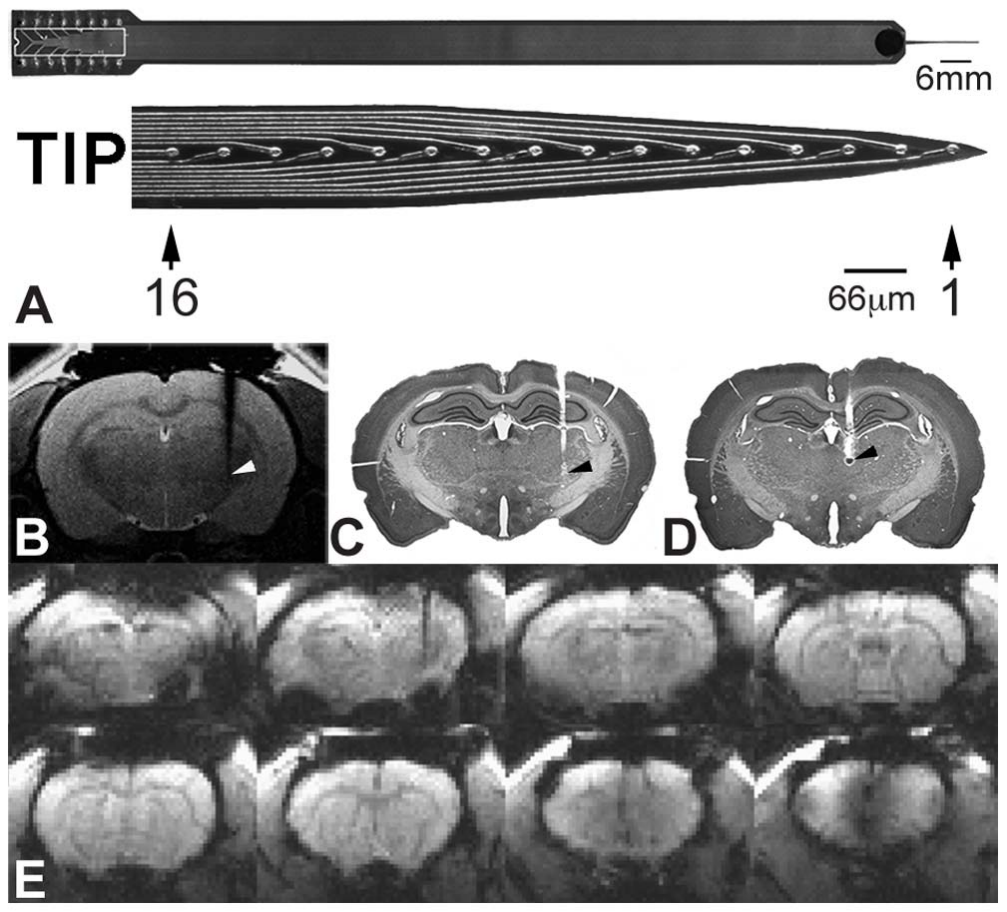
Stimulation electrode and its MR compatibility. Photograph of the electrode and tip (TIP) are displayed in (A). Coronal T2 image with the implanted electrode track in (B), Nissl-stained section showing the location of the electrode tip in the VP (C) and the MT (D). Eight coronal gradient-echo EPI images are presented from caudal to rostral (E).

Each animal was positioned in a stereotaxic frame and a small hole was drilled into its skull. A magnetic resonance (MR) compatible microelectrode array (Fig. 1A) was then implanted into the right VP (including the ventroposterior medial/ventroposterior lateral nuclei (VPM/VPL); 3 mm caudal to the bregma, 3 mm from the midline, and 6 mm ventral to the pia surface) or the right MT (2.5 mm caudal to the bregma, 0.9 mm from the midline, and 5.1 mm ventral to the pia surface). The electrode had 16 evenly spaced electronic-stimulation/neural recording pads, and its specifications and fabricating procedure were as described previously [15], [16]. Briefly, the microelectrode array was bonded to a long polyimide-based cable with pins soldered into 2 rows of 8 pins in a wide dual-inline pin format. The microelectrode array was constructed with integrated connector pads, a long shaft, and recording sites evenly spaced at 66-µm intervals (Fig. 1A). The impedance of the 16 electrodes on the microelectrode array, as measured using impedance spectroscopy, was approximately 250 kΩ at 100 Hz and 800 kΩ at 1 kHz. The induced voltage from the electrodes was measured during MRI scanning using multimeter and oscilloscope (TDS2004B, Tektronix, Beaverton, OR, USA). The maximum induced voltage was approximately 5 mV. According to the electrode impedance and the Ohm’s law, the maximum calculated value of the current induced by rapidly changing magnetic fields was approximately 0.02 µA. As shown in Fig. 1B, the electrode tract was clearly visible on MR coronal images of the rat brain. These MR images, which included the implanted microelectrode array, revealed low susceptibility artifacts. The tract and location of the electrode tip for stimulating the VP (Fig. 1C) and MT (Fig. 1D) were further confirmed in histological sections.

The electrode was fixed to the rat skull using dental cement and covered with a layer of 2% agar. The rectal temperature was then measured using a thermocouple and maintained at 37±0.5°C by a feedback-controlled blanked system (Harvard Apparatus, Holliston, MA, USA). A head-stage amplifier with a gain of 20 (Plexon, Dallas, TX, USA) was used to amplify neural signals that were then sent to a preamplifier. Signals were filtered at 154 Hz to 13 kHz with a gain of 50. Multichannel signals were recorded using a Multichannel Acquisition Processor (Plexon) for on-line spike sorting, acquisition (with a 12-bit analog to digital (A/D) conversion and a 40-kHz sampling rate in each channel), and amplified signals (variable gains, with maximal gains of up to 30000). Multiunit signals were post-processed using Offline Sorter (Plexon) for off-line spike sorting (to distinguish single units from multiunit clusters). NeuroExplorer (Nex Technologies, Littleton, MA, USA) was used for neurophysiological data analysis. The receptive fields of sensory neurons in the VP or MT were identified in different channels according to their responsiveness to peripheral air-puff stimuli and pinching.

### Thalamic Stimulation Paradigms

A constant electrical current with a pulse width of 0.4 ms was delivered through the MR-compatible microelectrode array to the VP or MT by an isolated stimulator (S48 Square Pulse Stimulator with PSIU6 Isolation Units, Grass Technologies, West Warwick, RI, USA). The intensity and frequency of the electrical stimulation was adjusted in values ranging from 50 µA to 300 µA and 1 Hz to 12 Hz, respectively, and consisted of 5 trains of 20 s duration interleaved with 20 s without stimulation. We applied various intensities and frequencies randomly and 5–10 min inter-session interval was allowed between the trials.

**Figure 2:**
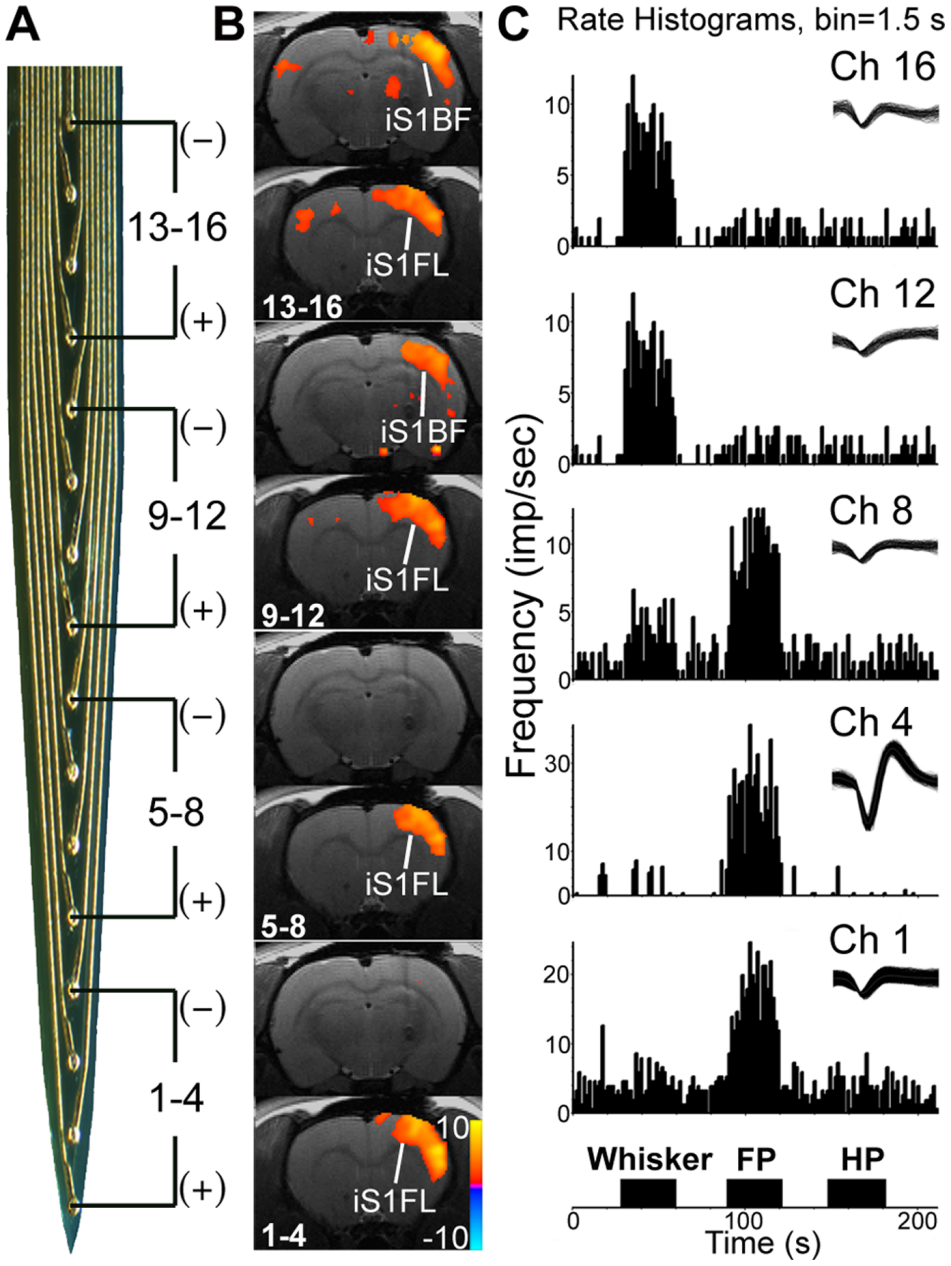
VP stimulation evoked BOLD activation maps that relate to sensory representation. (A) The four electrode-pad pairs that were used for bipolar constant current stimulation (3 Hz, 200 µA) are diagrammatically shown on the photomicrograph of the linear 16-channel stimulation electrode. (B) BOLD activation maps. Two BOLD maps, one related to the rostral iS1BF and the other caudal iS1FL levels, are shown for each of the four electrode pairs. (C) Neural responses in the VP recording by the microelectrode array. Air-puff stimulation was used on rat including forepaw (FP), hind-paw (HP) and whisker.

Bipolar stimulation of the thalamus was delivered between 2 electrode pads in the array so that the anode was always the deepest electrode tip and the cathode was located in the dorsal region of the electrode (Fig. 2A). Not all pairs could produce good BOLD activation. Therefore, we need to change the pair of electrodes for effective stimulation. For example, if channels 1 and 4 did not evoke good activation in the brain, another channel pairs (for example, channels 2 and 5) would be used for stimulation. The starting pair was randomly selected. The 2 contact pads were separated with a inter-electrode distance of either 198 or 462 µm.

### Functional MRI Acquisition and Data Processing

The fMRI experiments were performed in a 7-Tesla scanner with a 30-cm diameter bore (Bruker Biospec 7030 USR, Ettlingen, Germany. The system was equipped with a 670-mT/m (175-µs rise time) actively shielded gradient system (Bruker, BGA12-S) with an inner diameter of 116 mm. A 4-element phased array coil was used to receive the radio frequency signal and a linear volume coil was used to transmit the radio frequency pulses. The animal and the surface coil were secured in a custom animal holder (Bruker). This holder contained a blanket system to circulate warm water, maintaining the rat body temperature at 37°C. A pressure sensor (SA Instruments, Inc., NY, USA) was positioned under the abdomen of the rat to monitor respiration (maintained at 45 breaths/min to 55 breaths/min). The T2-weighted images were obtained using a rapid acquisition with refocused echoes (RARE) sequence (TR = 2500 ms, TE = 33 ms, matrix size = 156×156, field of view (FOV) = 25 mm×25 mm, average = 2). Before functional image scanning, a shim calculation for the brain area was applied to improve the magnetic field homogeneity (MAPSHIM, Bruker). Functional images were created using single-shot gradient-echo echo planar imaging (GE-EPI) of 10 coronal rat brain slices of 1 mm thickness (FOV = 25×25 mm, matrix size = 80×80, TR = 2000 ms, TE = 20 ms, bandwidth 200 kHz). In a boxcar design, each session consisted of 110 scan images, including 5 stimuli blocks and 6 control blocks (one block consisted of 10 images: 20 s). An example of an image from GE-EPI acquired during VP-implanted experiments is shown in Fig. 1E.

The 110 EPI images from the thalamic stimulation experiments were registered to an anatomical image using a linear image registration tool (FLIRT, FSL). Activation maps were created using a cross-correlation function with a time course at each voxel, using a boxcar design of the stimulation protocol (3dDeconvolve, AFNI). Group analysis of individual statistical maps was performed using Student’s t-test (3dttest++, AFNI). Significant pixels in the EPI images were detected at an individual pixel threshold of *p*<0.01 (with a corrected false discovery rate) and a minimum of 5 continuous pixels. Results of the activation maps are displayed as statistical t-value maps: warm-colored activation maps indicate increases in BOLD and cold-colored activation maps indicate decreases in BOLD. The statistical threshold activation map was then superimposed on the T2-weighted images.

### Analysis of Regions of Interest (ROI)

To obtain average BOLD time courses from the series of EPI images from each rat in the ipsilateral and contralateral cingulate cortex (iCC and cCC), MT, ipsilateral and contralateral forelimb region of the primary somatosensory cortex (iS1FL and cS1FL), ipsilateral barrel field of the primary somatosensory cortex (iS1BF), and ipsilateral and contralateral caudate-putamen (iCPu and cCPu), we performed conventional ROI analysis. A rat atlas was used for guidance when selecting the dimensions and locations of the polygonal ROI [17]. All pixels were averaged within the ROI in each image. The response amplitude was calculated from the 5 stimulus blocks. Statistical data were analyzed using a one-way analysis of variance (ANOVA) followed by the Tukey’s method for multiple comparisons, and a 2-way repeated measurement ANOVA followed by the Newman-Keuls method for multiple comparisons. A *p-*value <0.05 was considered significant. Region of interest analytical data are presented as the mean ± standard error of the mean (SEM) unless stated otherwise. The % changes in the BOLD signals were calculated by normalizing the stimulation blocks to the control blocks (first 20 s control block).

### Histology

After collecting the MRI scans, the rat was sacrificed using sodium pentobarbital overdose (100 mg/kg IV) and perfused transcardially with warm saline followed by 10% formalin. The brain was removed, stored overnight, and cut into serial coronal sections (50 µm). The sections were then processed for Nissl staining (Figs. 1C and 1D).

### Immunohistochemical Analysis of c-Fos Expression

The MR-compatible microelectrode array implantation procedure was identical to that used for electrode implantation and recordings. Local anesthetics were applied to all wounds (EMLA: 2.5% lidocaine and 2.5% prilocaine; AstraZeneca, Södertälje, Sweden). Two hours after implantation of the electrodes (MT stimulation: 9 Hz, n = 3; 3 Hz, n = 3; MT sham: 9 Hz, n = 3; 3 Hz, n = 3; VP stimulation: 9 Hz, n = 3; 3 Hz, n = 3; VP sham, n = 3), 2 electrical stimulation periods (with an intensity of 200 µA) were applied to the MT and VP. The electrical stimulation procedure was identical to that used in the fMRI experiments (20 s on, 20 s off, repeated 5 times/period). Two hours after electrical stimulation, rats were deeply anesthetized using a high dose of sodium pentobarbital (100 mg/kg) and perfused transcardially with 0.1 M phosphate buffered saline (PBS) followed by 4% paraformaldehyde (Merck, Darmstadt, Germany) in 0.1 M PBS. After fixation, the brain was removed, postfixed in the same fixative for 24 h, and immersed in 30% sucrose (Sigma, St. Louis, MO, USA) in 0.1 M PBS at 4°C. Serial frozen sections (40 µm) were cut using a microtome. Sections were collected and rinsed with 0.1 M PBS and 0.3% hydrogen peroxidase for 30 min. They were then rinsed with 0.1 M PBS, blocked for 2 h with 3% normal goat serum and 0.2% Triton X-100 (Sigma), and incubated with a primary Fos protein antibody (sc-52, Santa Cruz Biotechnology, Santa Cruz, CA, USA), diluted 1∶1000 in 0.1 M PBS containing 3% normal goat serum and 0.2% Triton X-100 for 48 h at 4°C. After washing in 0.1 M PBS, sections were further incubated with a biotinylated secondary antibody (1∶500; Vector Laboratories, Burlingame, CA, USA) for 2 h at room temperature, and with the avidin-biotin peroxidase complex (Vectastain Elite ABC kit, Vector Laboratories) for 1 h at room temperature. Sections were visualized using a 0.05% diaminobenzidine (DAB, Sigma) solution with 1% nickel ammonium sulfate and 0.3% hydrogen peroxidase. Sections were mounted on glass slides coated with gelatin, air-dried, and dehydrated in 100% alcohol before application of the coverslips with DPX (Sigma). Four randomly selected and evenly spaced brain sections each, containing the iCC, cCC, iS1FL, cS1FL, iCPu and cCPu regions of each rat were photographed at 20× magnification using a CCD camera installed on a Zeiss microscope (Hallbergmoos, Germany). Numbers of c-Fos-labeled cells were counted using ImageJ software (Cell Counter plugins). Data on c-Fos-labeled cells are presented as the mean ± SEM. Statistical data analyses were performed using one-way ANOVA followed by Tukey’s method.

## Results

### Deep Brain Stimulation-evoked SI BOLD Responses Topographically Related to Receptive Fields of the VP

We recorded multi-units in the VP evoked by air-puff stimulation of the whiskers, forepaw (FP), and hind-paw (HP), and used the Plexon Offline Sorter to isolate a single unit from each of the 16 channels. Forepaw responsive units were predominantly represented in the deeper locations (channels 1 to 8), whereas whisker units were represented in shallow locations (channels 8 to 16). We were unable to identify any unit responsive to hind paw stimulation (Fig. 2C). According to these findings, we partitioned the 16 channels into 4 groups (bottom: channels 1 to 4; mid-bottom: channels 5 to 8; mid-top: channels 9 to 12; and top: channels 13 to 16; with an inter-electrode pad distance of 198 µm) for bipolar stimulation delivered between the anode and cathode (Fig. 2A). As shown in Fig. 2B, brain activation occurred in the iS1FL and iS1BF following direct VP stimulation (200 µA, 3 Hz). Different locations of stimulation evoked responses in different cortical areas. Bottom and mid-bottom locations of stimulation induced robust activation in the iS1FL. Mid-top and top locations of stimulation induced robust activation in the iS1FL and iS1BF. These results indicated that the responses evoked by VP stimulation were in accordance with the VP sensory map. We also tested the tactile- and pinch-receptive fields of the MT stimulation sites but were unable to identify any clear topography. We did not, therefore, further analyze this aspect.

### Mapping of the Lateral Thalamocortical Projections: the Effects of Stimulus Intensity

**Figure 3:**
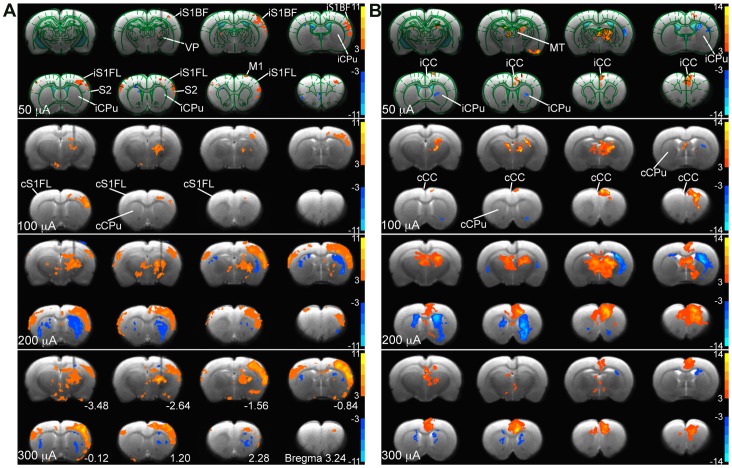
Intensity-dependent activity maps and region of interest analysis in the brain during VP and MT stimulation. The group activation maps elicited by VP (3 Hz, n = 4) and MT (9 Hz, n = 6) stimulation at four intensities level (from top to bottom, 50, 100, 200 and 300 µA) shown on eight coronal sections of the forebrain in two rows. (A) With VP stimulation, positive BOLD responses (warm color) were activated most strongly in the iS1FL and iS1BF, and negative BOLD responses (cold color) in the iCPu and cCPu. (B) With MT stimulation, positive BOLD responses were activated most strongly in the iCC, and most negative responses were found in the iCPu. Demarcations of region of interest (ROI) are outlined in the top panel of the figures.

**Figure 4:**
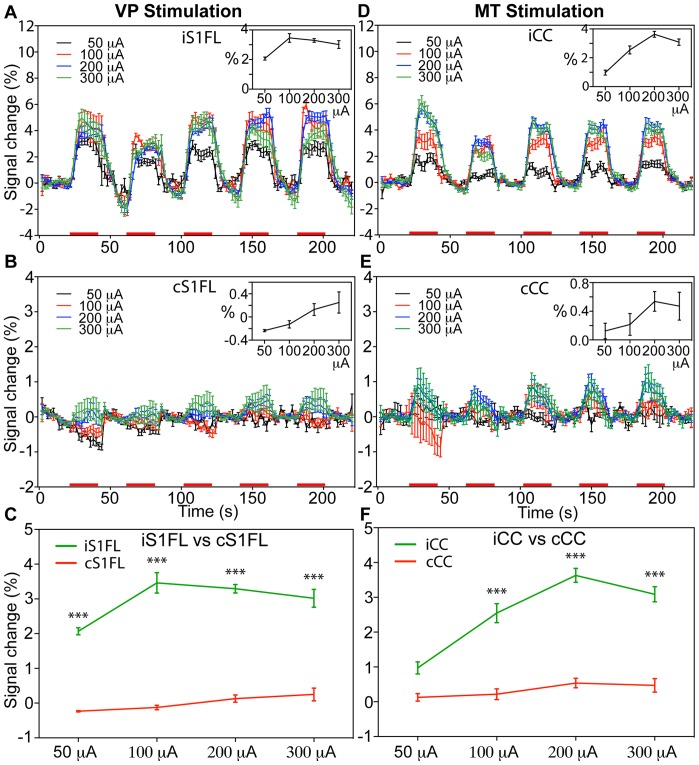
Comparison of BOLD signal changes in the cortical targets by various thalamic stimulation intensities. Mean time course (mean ± SEM, n = 4) of BOLD signal changes within the iS1FL (A) and cS1FL (B) at four stimulus intensities. Average time course (mean ± SEM, n = 6) of BOLD signal changes within the iCC (D) and cCC (E) at four stimulus intensities. Red bars under the time axis show the periods of electrical stimulation. The mean BOLD response amplitude (average change, %) within the five stimulus blocks is shown against the stimulus intensity in the inset and enlarged in (C) and (F). (*** *p*<0.0005, two-way ANOVA with Newman-Keuls posttest).

Direct VP stimulation using 4 different intensity stimuli (50, 100, 200, and 300 µA; 3 Hz, n = 4, with an inter-electrode pad distance of 462 µm) evoked steady-state strong positive BOLD responses (PBRs) in the iS1FL. Figure 3A shows the group activation maps evoked by various stimulus intensities in 8 coronal slices. At 50-µA and 100-µA stimulation, only a few BOLD activations were presented in the cortex. For 200-µA stimulation, the largest activated areas were detected in the iS1FL, the primary motor cortex (M1), the secondary sensory regions (S2), the iS1BF and reproducible negative BOLD responses (NBRs) in the bilateral CPu. We observed PBRs in the iS1FL in 3 consecutive slices, and in the iS1BF in 3 consecutive slices, in the anterior-posterior direction at 200-µA and 300-µA stimulation. Figure 4A displays the average time course within the iS1FL of 4 rats. The inset displays the mean PBR amplitude within 5 stimulus blocks plotted against the graded stimulus intensities. At stimulus intensities lower than 100 µA, we observed increasing PBRs amplitudes with increasing stimulus intensities, and reach a plateau above 100 µA. The PBRs elicited at 50-µA stimulus intensity were significantly lower than those elicited at intensities higher than 100 µA (*F* = 37.77, *p*<0.0001), while, the largest PBRs (3.46±0.29%) were detected when applying a 100-µA stimulus intensity. Moreover, there was no significant difference in activation amplitude among 100-, 200-, and 300-µA stimulation (*F* = 2.12, *p*>0.05). In the cS1FL areas, the PBRs amplitude shows a linear rise of the amplitudes from 50 to 300 µA, and the highest activations of 0.25±0.03% at 300 µA (Figure 4B). As Figure 4C clearly demonstrate, there was strongly significant difference for all stimulus intensities between iS1FL and cS1FL (*p*<0.0005, two-way ANOVA with Newman-Keuls posttest). Averaged BOLD amplitude of iS1FL was significantly higher than that in cS1FL.

### Mapping the Medial Thalamocortical Projections: the Effects of Stimulus Intensity

Direct MT stimulation at 4 different stimulus intensities (50, 100, 200, and 300 µA; 9 Hz, n = 6, with an inter-electrode pad distance of 462 µm) evoked steady-state and strong PBRs in the bilateral CC and MT. Figure 3B shows the group activation maps evoked at various intensities in 8 coronal slices. The activated areas were detected in the iCC, cCC and MT. We also identified reproducible NBRs in the iCPu and cCPu. At 50-µA and 100-µA stimulation, only a few BOLD activations were showed in the iCC, iCPu and MT. At 200 and 300 µA, we observed large areas of PBRs in 4 consecutive slices in the iCC, and in 2 consecutive slices in the MT, in the anterior-posterior direction And, we also detected a large area of negative activations in iCPu and smaller area in cCPu at 200-µA stimulation, and a small area of activation at bilateral CPu at 300 µA. Figure 4D displays the average time course within the CC of 6 rats, with the mean PBR amplitude within 5 stimulus blocks plotted against the stimulus intensity in the inset. We observed significant differences among BOLD signal changes elicited at 50 µA and those elicited at intensities >100 µA (*F* = 37.74, *p*<0.0001). We also found the largest BOLD signal change (3.63±0.20%) after applying a stimulus intensity of 200 µA: the BOLD amplitude was significantly greater than that at 50 µA (*p*<0.0001) and 100 µA (*p*<0.001). However, the differences between the BOLD amplitudes elicited at 200-µA and 300-µA stimulus intensities were insignificant (*p*>0.05). In the cCC areas (Figure 4E), We found a similar patterns in increased BOLD amplitude with increasing stimulus intensity below 200-µA stimulation as in the iCC. But, there was strongly significant difference between iCC and cCC at all stimulus intensities (*p*<0.0005, two-way ANOVA with Newman-Keuls posttest). Averaged BOLD amplitude of iCC was significantly higher than that in cCC.

### Mapping the Lateral Thalamocortical Projections: the Effects of Stimulus Frequency

**Figure 5:**
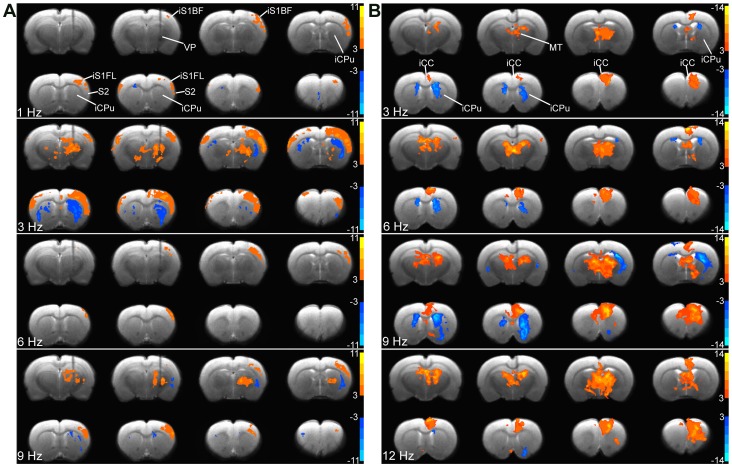
Frequency-dependent activity maps and region of interest analysis in the brain during VP and MT stimulation. The group activation maps evoked by VP (n = 6) and MT (n = 6) stimulation at various frequencies at 200 µA are shown on eight coronal sections of the forebrain in two rows from top left to lower bottom, caudal to rostral. (A) Note that VP stimulation most strongly activated BOLD responses within iS1FL at 3 Hz. (B) MT stimulation most strongly activated BOLD responses within iCC at 9 ∼ 12 Hz. MT stimulation at 1 Hz elicited no response.

**Figure 6:**
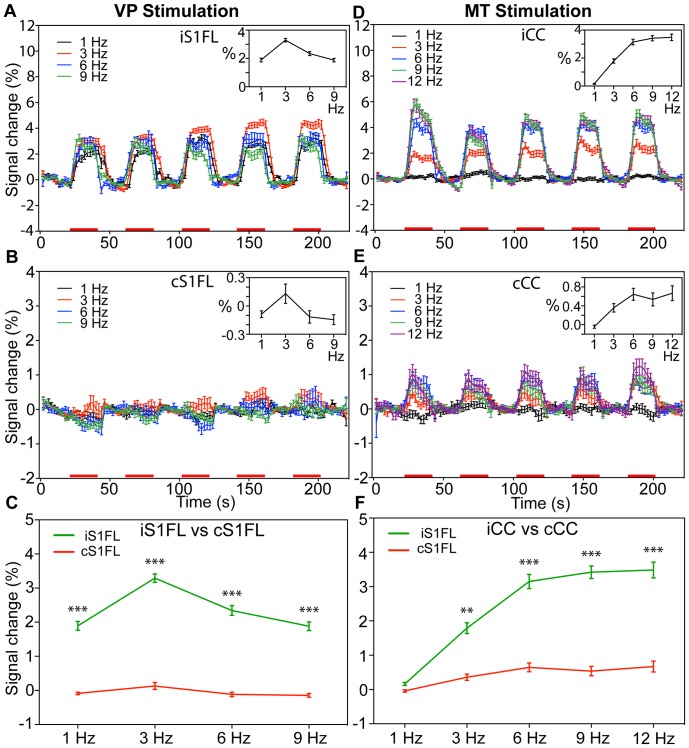
Comparison of BOLD signal changes in the cortical targets by various thalamic stimulation frequencies. Mean time course (mean ± SEM, n = 6) of BOLD signal changes within the iS1FL (A) and cS1FL (B) at four stimulus frequencies. Average time course (mean ± SEM, n = 6) of BOLD signal changes within the iCC (D) and cCC (E) at five stimulus frequencies. Red bars under the time axis show the periods of electrical stimulation. The mean BOLD response amplitude (average change, %) within the five stimulus blocks is shown against the stimulus intensity in the inset and enlarged in (C) and (F). (** *p*<0.001, *** *p*<0.0005, two-way ANOVA with Newman-Keuls posttest).

Direct VP stimulation induced reproducible PBRs in the iS1FL and iS1BF in all rats (n = 6); therefore, we selected a stimulus intensity of 200 µA (with an interelectrode pad distance of 462 µm) for analyses. Figure 5A shows the group activation maps evoked by various frequencies in 8 coronal slices. The large area of activations in the iCC and iCPu were detected at 3-Hz stimulation, while, the small area of activations were found at others stimulation. Figure 6A displays the average time course within the iS1FL of 6 rats. We observed significant differences among BOLD signal changes elicited by a 3-Hz frequency stimulus and those elicited at other stimulus frequencies (1, 6, and 9 Hz), the largest BOLD signal changes within the iS1FL (3.29±0.12%) and iS1BF (3.24±0.14%, data not shown) at 3 Hz. The BOLD amplitude was significantly larger than those evoked at other stimulus frequencies (*F* = 24.56, *p*<0.0001). In the cS1FL areas, the PBRs amplitude shows bell-shaped responses curves from 1 to 9 Hz, and the highest activations of 0.13±0.10% at 3 Hz (Figure 6B). As Figure 6C clearly demonstrate, there was strongly significant difference between iS1FL and cS1FL at all stimulus frequencies (*p*<0.0005, two-way ANOVA with Newman-Keuls posttest). Averaged BOLD amplitude of iS1FL was significantly higher than that in cS1FL.

### Mapping the Medial Thalamocortical Projections: the Effects of Stimulus Frequency

Direct MT stimulation produced reproducible PBRs in the iCC and MT in all rats (n = 6); therefore, we selected a stimulus intensity of 200 µA (with an inter-electrode pad distance of 462 µm) for analyses. Figure 5B shows the group activation maps evoked by various frequencies in 8 coronal slices. At a lower stimulus frequency (1 Hz), we did not observe significant brain activation (data not shown). At stimulus frequencies ≥3 Hz, we detected robust PBRs in the iCC and MT and NBRs in the iCPu and cCPu. Figure 6D displays the average time course within the iCC of 6 rats. We observed significant differences among the BOLD signal changes induced at lower (<3 Hz) and higher (>6 Hz) frequencies (*F* = 72.62, *p*<0.0001), and the largest BOLD signal change (3.48±0.22%) within the iCC at 12 Hz, with similar results within the ipsilateral MT (3.17±0.16%; data not shown), with the BOLD amplitude significantly larger than those induced at 1 Hz and 3 Hz (*p*<0.0001). However, at 6, 9, and 12 Hz, the differences among the elicited BOLD amplitudes were insignificant. In the cCC areas (Figure 6E), We found a similar dose-response curve. But, there was strongly significant difference for stimulus frequencies between iCC and cCC at ≥3 Hz (two-way ANOVA with Newman-Keuls posttest). Averaged BOLD amplitude of iCC was significantly higher than that in cCC.

### Functional Brain Activation Following VP and MT Stimulation

**Figure 7:**
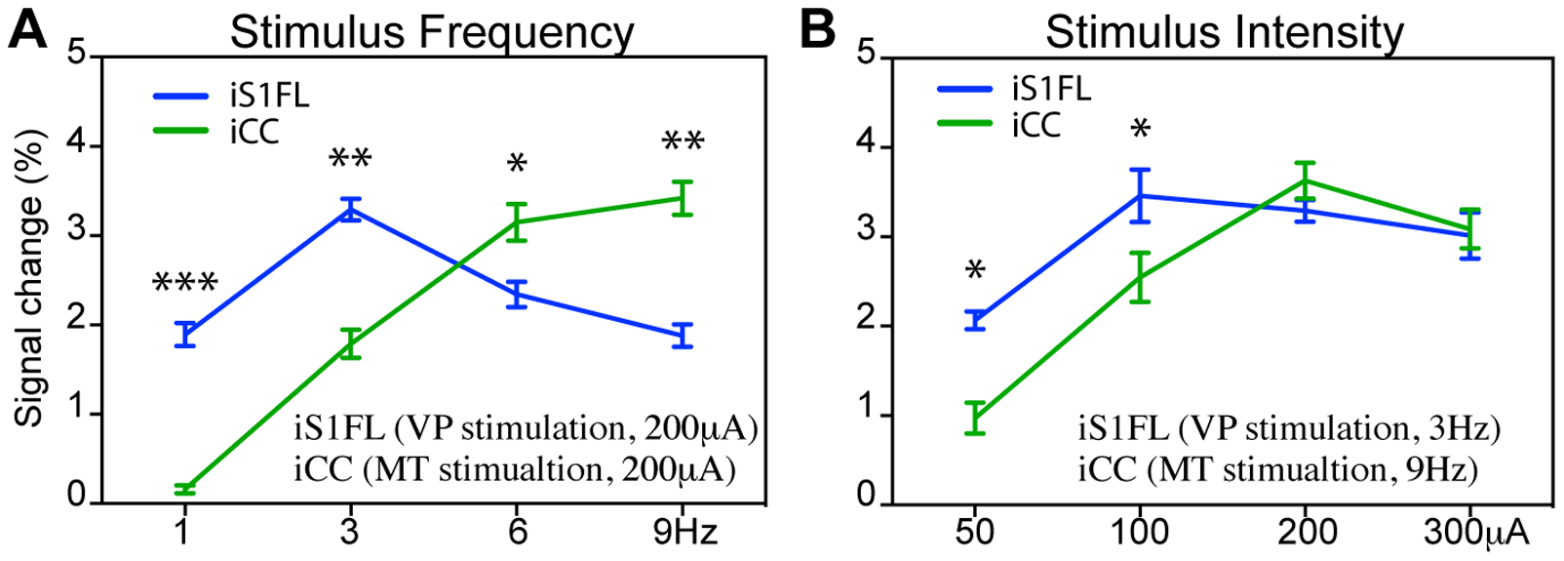
Comparison of evoked BOLD responses between VP and MT stimulation. Mean BOLD response amplitude (mean ± SEM) in the S1FL (VP stimulation; blue) and CC (MT stimulation; green) at (A) different frequencies (200 µA) and (B) intensities (VP: 3 Hz; MT: 9 Hz). Compared to VP stimulation, the MT required a stronger and higher stimulation frequency to produce an effect (* *p*<0.05, ** *p*<0.001, *** *p*<0.0005, two-way ANOVA with Newman-Keuls posttest).

To compare BOLD signals after VP and MT stimulation, we averaged the BOLD signals evoked throughout the stimulus duration. Figure 5 displays the summarized frequency- and intensity-dependent BOLD contrast changes elicited by VP and MT stimulation. Ventroposterior thalamic stimulation induced changes in the iS1FL at a low intensity (50 µA) and low frequency (1 Hz); however, this rapidly reached a maximal effect at 100-µA intensity and 3-Hz frequency. Medial thalamic stimulation induced changes in the CC that required higher intensity (200 µA) and higher frequency (6 Hz) stimulation to achieve the peak intensity. The effects in the VP-iS1FL had a bell-shaped frequency domain (Fig. 7A) or a saturated intensity domain (Fig. 7B). This markedly contrasted with the effects in the MT-iCC, in which the frequency and intensity domain BOLD signals showed graded response curves following VP and MT stimulation. These effects significantly differed between VP and MT stimulation (Fig. 7A, *F* = 22.16, *p*<0.0001, 2-way ANOVA of frequency level×region) and at all frequencies (2-way ANOVA with Newman-Keuls posttest), while, in different intensities not significantly difference (Fig. 7B, *F* = 0.198, *p* = 0.89, 2-way ANOVA of intensity level×region), but significantly differed at intensities of 100 µA to 200 µA (Fig. 7B, 2-way ANOVA with Newman-Keuls posttest).

### Negative BOLD Responses in the CPu

**Figure 8:**
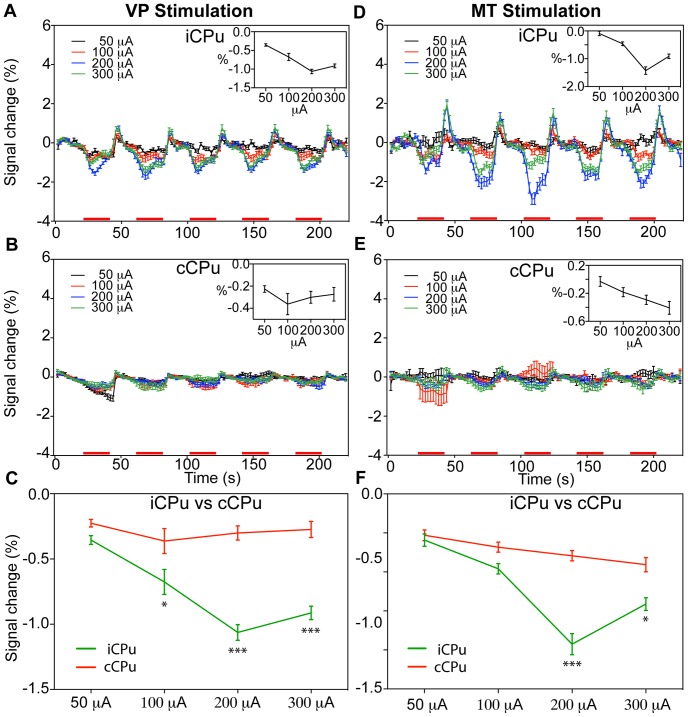
Comparison of negative BOLD responses in the CPu by thalamic stimulation intensities. Mean time course of BOLD responses (mean ± SEM) induced by VP (A, B; n = 4) and MT (D, E; n = 6) stimulation in the iCPu and cCPu, depicted for various stimulus intensities. Red bars under the time axis show the periods of electrical stimulation. The mean BOLD response amplitude (average change, %) within five stimulus blocks is shown against the stimulus intensity in the inset and composited in (C) and (F). (* *p*<0.05, *** *p*<0.0005, two-way ANOVA with Newman-Keuls posttest).

**Figure 9:**
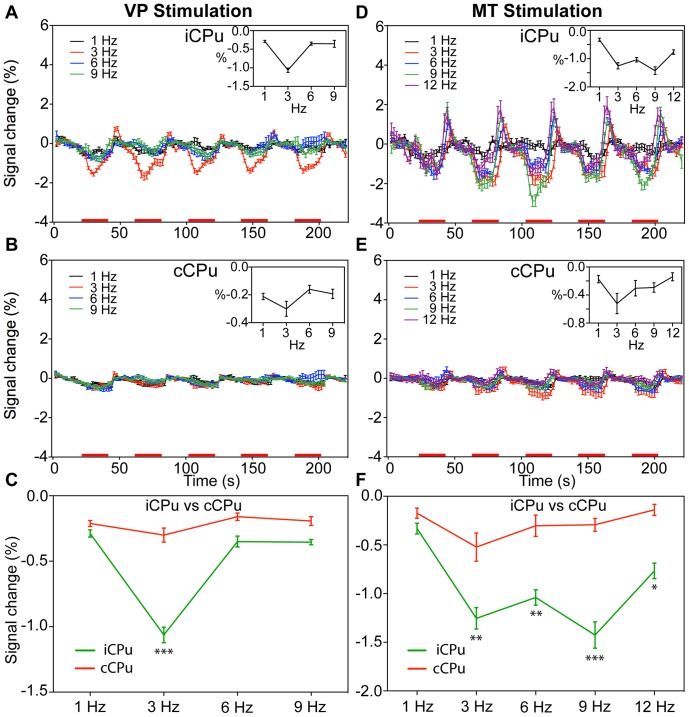
Comparison of negative BOLD responses in the CPu by thalamic stimulation frequencies. Mean time course of BOLD responses (mean ± SEM) induced by VP (A, B; n = 4) and MT (D, E; n = 6) stimulation in the iCPu and cCPu, depicted for various stimulus frequencies. Red bars under the time axis show the periods of electrical stimulation. The mean BOLD response amplitude (average change, %) within five stimulus blocks is shown against the stimulus frequency in the inset and composited in (C) and (F). (* *p*<0.05, ** *p*<0.001, *** *p*<0.0005, two-way ANOVA with Newman-Keuls posttest).

We detected NBRs in the bilateral CPu following VP stimulation (Figs. 3A and 5A) and MT stimulation (Figs. 3B and 5B). Figure 8 and 9 displays the average time course within the iCPu using different currents and frequencies. The insets show the BOLD response amplitudes versus the stimulus intensities and frequencies. When stimulating the VP and MT, the NBRs of the iCPu were weak at 50 µA and 100 µA stimulus intensities (Fig. 8A and 8D). We detected the highest NBRs amplitude at 200-µA VP stimulation (−1.06±0.05%, n = 4) and MT stimulation (−1.43±0.14%, n = 6). We also found a few NBRs that evoked by VP (Fig. 8B) and MT (Fig. 8E) stimulation in the cCPu. For VP and MT stimulation, the highest NBRs of the cCPu at 100 µA (VP, −0.36±0.09%) and 300 µA (MT, −0.41±0.09%). The amplitudes of NBRs were higher in the iCPu than those in the cCPu. In the VP stimulation (Fig. 8C), there was significant difference at 100 µA (*p*<0.05), 200 µA (*p*<0.0005) and 300 µA (*p*<0.0005), at 200 µA (*p*<0.0005) and 300 µA (*p*<0.05) for MT stimulation (Fig. 8F).

We evoked the CPu responses using a 200-µA current at 4 frequencies or VP stimulation and at 5 frequencies for MT stimulation. The NBRs of the iCPu had lower signal to noise ratio values because of the weak response following VP stimulation at 1, 6, and 9 Hz (Fig. 9A) We detected the highest NBRs amplitude value at 3 Hz (−1.06±0.06%, n = 4, *p*<0.05). When stimulating the MT, 1 Hz produced a very weak response, whereas 3, 6, 9, and 12 Hz (Fig. 9D) produced stronger responses. We detected the highest NBRs amplitude value at 9 Hz (−1.43±0.14%, n = 6, *p*<0.05) and observed a post-stimulus BOLD overshoot within the iCPu following both VP and MT stimulation. In general, BOLD responses in the iCPu following lower current (50 µA) or frequency (1 Hz) VP or MT stimuli were largely undetectable. NBRs elicited in the cCPu had relatively low amplitudes by VP stimulation (Fig. 9B) and MT stimulation (Fig. 9E). Compared NBRs in the iCPu and cCPu, there were significant differences at 3 Hz (*p*<0.0005, two-way AOVA with Newman-Keuls posttest) following VP stimulation (Fig. 9C), and at 3 Hz (*p*<0.001), 6 Hz (*p*<0.001), 9 Hz (*p*<0.0005) and 12 Hz (*p*<0.05) following MT stimulation (Fig. 9F).

### Immunohistochemical Analysis of c-Fos Expression

**Figure 10:**
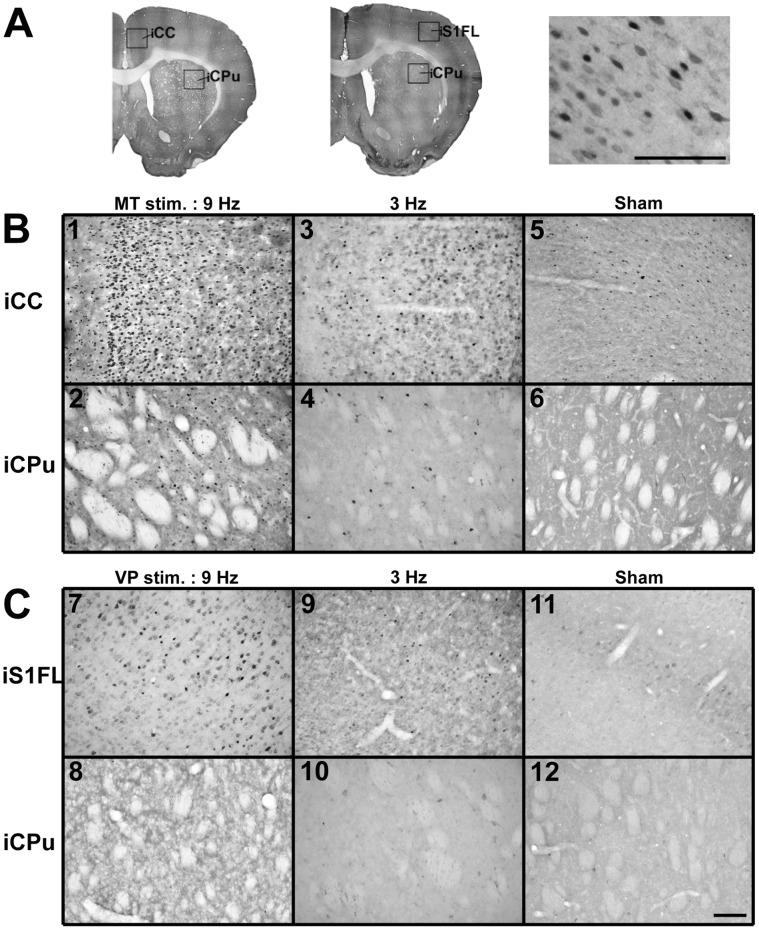
Photomicrographs depicting the distribution of Fos-immunoreactive cells in the brain regions of the iCC, iS1FL and iCPu after MT or VP stimulation. (A) Regions of interest where c-Fos labeled cells were counted. An example of the c-Fos labeled cells is shown in the right panel. (B) Show examples of the c-Fos labeled cells within iCC and iCPu with a 9-Hz (1,2) MT stimulation compare to 3-Hz stimulation (3,4) and sham control (5,6). (C) Examples of c-Fos labeled cells within the iCC and iCPu with 9-Hz (7,8) VP stimulation compared to 3-Hz stimulation (9,10) stimulation and the sham control (11,12). Scale bar = 0.1 mm.

Stimulation of the right MT (Fig. 10B) and VP (Fig. 10C) induced c-Fos expression in the iCC, iS1FL, and iCPu. We observed c-Fos-labeled cells, as indicated by dark spots of immunolabeling, in the ipsilateral hemisphere (Fig. 10A, right). Following 9-Hz right MT stimulation, we identified large numbers of c-Fos-positive cells in the iCC (Fig. 10B-1) and iCPu (Fig. 10B-2). Following 3-Hz right MT stimulation, however, we detected few c-Fos-labeled cells in the iCC and iCPu (Figs. 10B-3 and 10B-4) and in the sham control group (Figs. 10B-5 and 10B-6). Stimulation of the right VP elicited c-Fos expression in few scattered cells in the iS1FL and iCPu at 9 Hz (Figs. 10C-7 and 7C-8), and in rare cells in the same regions at 3 Hz (Figs. 10C-9 and 10C-10) and in the sham control group (Figs. 10C-11 and 10C-12). Table 1 displays the c-Fos-positive cell counts within the ROI in the iCC and iS1FL, and iCPu (Fig. 10A, left and middle). Stimulation of the right MT elicited significantly higher c-Fos expression in the iCC and iCPu at 9 Hz than at 3 Hz (*p*<0.0001) and compared to the MT sham control group (*p*<0.0001). We observed insignificant differences in c-Fos expression in the iCC and iCPu between the 3-Hz MT stimulation and the MT sham control groups. We observed a significantly higher number of c-Fos-positive cells in the iS1FL following application of 9-Hz VP stimulation than after 3-Hz VP stimulation (*p*<0.05) or compared to the VP sham control group (*p*<0.0001). However, the differences in c-Fos expression in the iS1FL between the 3-Hz VP stimulation and VP sham control groups were insignificant. In addition, there were insignificant differences in c-Fos-labeling in the iCPu among the 9-Hz VP stimulation, 3-Hz VP stimulation, and the VP sham control groups (*p*>0.05). We observed few scattered c-Fos-labeled cells in the contralateral hemisphere, including the cCC (except after 9-Hz MT stimulation), cS1FL, and cCPu.

**Table 1:** c-Fos cells after MT and VP stimulation.

	MT stimulation		MT Sham		VP stimulation		VP Sham
	9 Hz	3 Hz			9 Hz	3 Hz	
iCC	456.0±22.2	14.8±1.3	5.2±2.0	iS1FL	9.3±1.7	4.1±1.1	0.9±0.2
cCC	87.8±7.7	18.4±1.3	7.7±3.2	cS1FL	0.3±0.1	2.0±0.6	1.0±0.6
iCPu	115.3±16.2	2.3±0.5	0.3±0.2	iCPu	1.1±0.1	1.4±0.2	1.0±0.3
cCPu	3.1±0.8	2.3±0.5	0.7±0.2	cCPu	0.3±0.1	0.2±0.1	0.3±0.1

Data represented as mean ± SEM (number/mm^2^). MT, medial thalamus; iCC, ipsilateral cingulate cortex; cCC, contralateral cingulate cortex; iCPu, ipsilateral caudate putamen; cCPu, contralateral caudate putamen; VP, ventral posteromedial/posterolateral nucleus; iS1FL, ipsilateral forelimb region of primary somatosensory cortex; cS1FL, contralateral forelimb region of primary somatosensory cortex.

## Discussion

Using 2 global brain imaging methodologies, this study compared the effects of direct stimulation of the VP of the lateral thalamic pathway and the MT of the medial thalamic pathway. We observed differential response patterns in their respective major cortical targets. These differences included: (1) lower threshold and earlier maximal BOLD effects as well as minimal c-Fos expression in the S1 resulting from VP stimulation, and (2) graded BOLD effects and marked increases in c-Fos expression in the CC resulting from MT stimulation. Stimulus-response function evaluates quantitative responses to quantitative stimuli. The shape of the stimulus response curve reveals the operational principles of the brain circuitry tested. The results in the present study indicated fundamental differences in responsiveness to direct stimulation and subsequent synaptic processing properties between medial and lateral thalamic pain pathways in our experimental condition.

The magnitude of the BOLD response in the S1 was highest at a VP stimulus frequency of 3 Hz, achieving a maximal response at 100 µA (Fig. 5, 6). Previous studies have extensively investigated the frequency- and intensity-dependent S1 BOLD responses to forepaw stimulation in the rat [18]–[22]. Our finding that the optimal stimulus frequency was 1.5 Hz to 3 Hz during α-chloralose anesthesia is similar to the observations reported previously. Several studies also described close association between neural activities (unit activity and field potential) and BOLD responses [19], [23]. These studies showed that both BOLD and neural responses rapidly achieved peak values at a stimulus frequency of 3 Hz, and that the responses gradually decreased with increasing frequency (up to 15 Hz). The optimal conditions for the S1 BOLD response to whisker stimulation depended on the anesthetics used and the type of stimulus. Two studies described that whisker stimulation, using a naturalistic stimulus of 7 Hz to 12 Hz, evoked the largest BOLD responses in the S1BF [22], [24]. Other studies reported that VPM responses to whisker stimulation were suppressed at frequencies higher than 2 Hz [25] and the optimal frequency for S1 response to direct electrical stimulation of the trigeminal nerve was 1 Hz to 3 Hz [26].

A BOLD response can be detected in the anterior cingulate cortex (ACC) of anesthetized rats subjected to intense electrical stimulation or noxious stimulation of the forepaw [27], [28]. To our knowledge, only one previous study reported the BOLD response in the rat cingulate cortex, resulting from a direct medial thalamic stimulus frequency of 10 Hz [11]. This study’s results support the described observations. We tested additional stimulus frequencies and intensities, observing that the magnitude of the BOLD responses gradually increased with increasing frequency (up to 12 Hz) and intensity.

Several studies have electrophysiologically evaluated cortical responses to direct thalamic stimulation in the medial thalamic and lateral somatosensory pathways. Their results showed some similarities and dissimilarities. Activation of both thalamocortical pathways produced a monosynaptic excitatory postsynaptic potential followed by a prolonged inhibitory postsynaptic potential. In contrast, paired-pulse stimulation usually elicited facilitation in the MT-ACC pathway [29] and inhibition in the lateral VP-S1 pathway [30], [31]. Repeated stimulation of the medial thalamic structures, such as the intralaminar and midline thalamic nuclei, at a frequency of 6 Hz to 12 Hz, produced a cortical recruiting response in which the widespread cortical response progressively increased with each subsequent stimulus in a train of stimuli [32]. This did not occur with VP stimulation [33].

Investigators have traditionally attributed the cause of the widespread cortical recruiting response to the diffuse and widespread thalamocortical projections of the medial thalamic nuclei. This study’s c-Fos data support this interpretation. More recent anatomical data, however, show that different medial thalamic structures have dense and specific connections with their specific cortical targets. This study focused on analyzing the rostral region of the ventral junction between the medial (MDm) and lateral (MDl) subnuclei of the medial dorsal nucleus. Assuming a diameter of influence from the stimulation center of 1 mm [34], [35], a 100-µA to 200-µA electrical stimulus would activate the entire MD, most of the rostral ILN, the paraventricular nucleus of the thalamus (PVT), other rostral midline structures, and probably areas of the anterior medial (AM) and anterior ventral (AV) nuclei. The directly involved cortical areas included the anterior cingulate cortex from the MDl, ILN, and AM, the prelimbic cortex from the MDm and midline thalamus, the infralimbic cortex from the PVT, the insular cortex from the MDm and ILN, and the motor cortices from the ILN [36]–[39]. Although all these areas showed c-Fos positivity, only the ACC and middle cingulate cortex (MCC) demonstrated a consistent and significant BOLD response. In our opinion, these conflicting results reflect the differences between a long-lasting but static activation pattern revealed by c-Fos expression and a dynamic hemodynamic activation pattern identified by the functional MRI methods. Using a block activation design, the BOLD response probes the dynamic response in the brain that correlates significantly with the pattern of the stimulation. The MT-CC pathway was the only thalamocortical pathway that responded dynamically and statically by a medial thalamic stimulation. Considered this way, our present result provides further evidence of the importance of the MT-CC pathway, shown in our previous study to have selective nociception-related manganese transportation [5].

In our lateral thalamus stimulation group, the same stimulus activated the VP only. The global pattern of the VP stimulation-induced BOLD effect showed strongest activation in the ipsilateral S1 and S2, with some activation in the contralateral S1, and reflected the strongest monosynaptic and disynaptic thalamocortical connections arising from the VP. Interestingly, only few cortical neurons showed cFos positivity, and these neurons were clustered only in the ipsilateral SI. The low-threshold, bell-shaped BOLD response and the sparsely but topographically located c-Fos response in the S1 elicited by VP stimulation might be necessary properties for a discriminatory system.

Previous research identified that the VP-S1/S2 is the main nociceptive pathway involved in sensory discrimination aspects of pain [1], [40]. Approximately 10% of its neurons are nociceptive and most are of the wide dynamic range type in the monkey [41], [42]. In the rat, approximately 50% of its neurons are nociceptive [43]. Nociceptive- and tactile-related neurons are intermingled in the core of the VP in both rats and monkeys. Nociceptive neurons in the VP are excited by noxious or innocuous stimuli [44]. These neurons often have small contralateral receptive fields that are organized somatotopically [45], [46]. In the MT, nociceptive neurons are excited by noxious mechanical [47], [48] and noxious visceral [49] stimuli, but are usually unresponsive to innocuous tactile stimuli. In addition, MT neurons usually have a large receptive field, without somatotopical organization, and are often bilateral. Human imaging studies further showed the ACC to participate in the affective component of pain [50], [51]. A behavioral and single unit study of the rat provided similar findings [3]. Functional distinction of the 2 thalamocortical pathways investigated in this study might facilitate the elucidation of the cellular mechanisms involved in different aspects of pain function.

In this study, stimulation of the VP or MT elicited consistent NBRs in the basal ganglia region. Previous study have reported the NBR in the vicinity of the PBR, it may be collocated the blood flow to activated regions from surrounding regions that possible statement of NBR is blood stealing effect [52]. However, Shmuel et al. [53] showed that NBRs were closely associated with decreases in neural activity in the human visual cortex. Northoff et al. [54] also reported that GABA concentration correlated with NBRs in the human ACC. These finding telling us positive correlation of NBR with neural suppression, but it still lack direct evidence. In nonhuman primates, simultaneous recording of BOLD and neural activities confirmed that NBRs predominantly correlated with neural inhibition [55]. However, other studies showed increased neuronal activity in regions with NBRs, such as the basal ganglia [56] and the hippocampus [57]. Our experiments might provide an independent model for further investigation of these inconsistent results.

In summary, this study demonstrated that electrical stimulation of the lateral and medial thalamus activated distinct target regions in the forebrain. Ventroposterior thalamic stimulation largely activated the S1, whereas stimulation of the medial thalamus activated the CC. Electrical stimulation of the VP and MT produced BOLD responses that were dependent on the intensity and frequency of the stimulus. The low-threshold and low-frequency characteristics of the effects in the VP may associate with its functions in the coding of the sensory-discrimination aspects of pain. On the other hand, the need for spatial and temporal summation to induce a response from the MT and the wide-spread and long-lasting c-Fos activation response is consistent with the motivational-affective functions of pain.
